# Immune therapies against chronic hepatitis B

**DOI:** 10.1007/s00535-022-01890-8

**Published:** 2022-06-16

**Authors:** Sheikh Mohammad Fazle Akbar, Osamu Yoshida, Yoichi Hiasa

**Affiliations:** Department of Gastroenterology and Metabology, Ehime University Graduate School of Medicine, Shitsukawa 454, Toon City, Ehime 791-0295 Japan

**Keywords:** Immune therapy, Chronic hepatitis B, Polyclonal immune modulators, Antigen-specific immunotherapy, Combination therapy

## Abstract

Patients with chronic hepatitis B (CHB) represent a living and permanent reservoir of hepatitis B virus (HBV). Millions of these CHB patients will eventually develop complications such as liver cirrhosis, hepatic failure, and hepatocellular carcinoma if they are not treated properly. Accordingly, several antiviral drugs have been developed for the treatment of CHB, but these drugs can neither eradicate all forms of HBV nor contain the progression of complications in most patients with CHB. Thus, the development of new and novel therapeutics for CHB remains a pressing need. The molecular and cellular mechanisms underlying the pathogenesis of CHB indicate that immune dysregulations may be responsible for HBV persistence and progressive liver damage in CHB. This provided the scientific and ethical basis for the immune therapy of CHB patients. Around 30 years have passed since the initiation of immune therapies for CHB in the early 1990s, and hundreds of clinical trials have been accomplished to substantiate this immune treatment. Despite these approaches, an acceptable regimen of immune therapy is yet to be realized. However, most immune therapeutic agents are safe for human usage, and many of these protocols have inspired considerable optimism. In this review, the pros and cons of different immune therapies, observed in patients with CHB during the last 30 years, will be discussed to derive insights into the development of an evidence-based, effective, and patient-friendly regimen of immune therapy for the treatment of CHB.

## Introduction

Hepatitis B virus (HBV) is a DNA virus of the hepadnaviridae family, and it infects humans of all ages. The virus is hepatotropic in nature and usually replicates in the hepatocytes, although HBV replication may take place in other tissues, including immunocytes. The replication system of the HBV in the hepatocytes is designed in such a way that the replicating forms of HBV DNA can be detected in the peripheral blood of HBV-infected subjects. In addition, an exceptional type of HBV DNA, covalently closed circular DNA (cccDNA), is formed in the nucleus of the hepatocytes as an episomal microchromosome. The cccDNA is a mighty form of HBV DNA and is capable of acting as a template for the transcription of new viral RNAs and HBV DNA [1, 2].

Epidemiologically, the HBV infection is global in nature, and it is estimated that HBV has infected about 2 billion people worldwide at some point in their lives. However, less than 15% of the total HBV-infected population (about 296 million out of 2 billion) are regarded as chronically infected by the HBV, and these patients express the hepatitis B surface antigen (HBsAg) in their blood for more than 6 months. Most of them also harbor HBV DNA in their blood in addition to expressing antibodies to the hepatitis B core antigen (HBcAg) (anti-HBc), the hepatitis B e antigen (HBeAg), or the antibody to HBeAg (anti-HBe). All chronic HBV-infected subjects are permanent and living reservoirs of the HBV, and they are primarily responsible for an estimated 1.5 million new HBV infections each year [3].

In addition to being living and permanent reservoirs of HBV infection, a considerable proportion of the estimated 296 million chronic HBV-infected subjects would eventually develop complications such as cirrhosis of the liver (LC), liver failure, and hepatocellular carcinoma (HCC). The World Health Organization (WHO) has estimated that there were about 820,000 deaths due to HBV-related liver diseases in 2019, and these were mainly due to HBV-related LC and HCC [3]. Interestingly, HBV-infected persons who develop LC or HCC or experience liver failure usually pass through stages of progressive hepatic inflammation (assessed by an increase in alanine aminotransferase [ALT]) with variable levels of progression of hepatic fibrosis. This intermediate stage of chronic HBV infection is manifested through the presence of HBsAg in the sera for more than six months, HBV DNA in the blood, elevated levels of ALT in the blood (indicating damage and destruction of hepatocytes), and features of variable levels of hepatic fibrosis. These patients are regarded as patients with chronic hepatitis B (CHB), as active therapies are recommended for them to block progression to LC and HCC [4]. Thus, HBV-related complications such as LC and HCC and about one million HBV-induced deaths can be mostly contained if efficient therapeutic modalities can be developed to treat and manage the condition of CHB patients [5].

As CHB is a virus (HBV)-induced pathological lesion of the liver, the primary attention in the treatment of CHB patients was targeted towards the reduction or clearance of HBV DNA with a postulation that viral reduction would be followed by the containment of liver damages and progression of HBV-related complications. Accordingly, several antiviral drugs were used on CHB patients for 3–4 decades. Eminent international professional liver organizations such as the American Association for the Study of Liver Diseases (AASLD) [6], the European Association for the Study of the Liver (EASL) [7], and the Asia–Pacific Association for the Study of the Liver (APASL) [8] have provided detailed recommendations for treating CHB patients on the basis of serum levels of HBV DNA, HBeAg, and ALT and histological status of liver damage.

As of today, two parental formulations of alpha interferon (standard and pegylated) and five oral medications of nucleos(t)ide analogs (NUCs, lamivudine, adefovir, entecavir, telbivudine, and tenofovir) have been approved by the Food and Drug Administration (FDA) of the United States of America (USA) for treating CHB patients. It is now apparent that commercially available antiviral drugs provide some beneficial effects in some CHB patients [9, 10]. However, these drugs are endowed with significant limitations that include

1. Considerable adverse effects,
2. Cost of the drugs for prolonged usage,
3. Cost and mode of administration (in cases such as interferon), and
4. Infinite usage (for NUCs).

In addition to these limitations, these drugs show inconsistent therapeutic efficacy in most CHB patients. Taken together, patients with CHB cannot usually expect a drug-free life and complication-free future after the cessation of the consumption of most of these antiviral drugs [11–14].

Thus, there is a pressing need to develop new and novel therapeutics for CHB treatment, either for their independent usage or for their use in combination therapy with other drugs. This has led to the creation of several lines of innovative medicines such as

1. Entry inhibitors,
2. Core protein allosteric modulators,
3. Drugs targeted for RNA interference,
4. Inhibitors of cccDNA, and
5. Therapeutic targeting cccDNA destruction [reviewed in detail in Reference 15].

Moreover, several lines of unique concepts have been formulated for treating CHB patients [16]. However, most of these innovative therapies are yet to be evaluated in CHB patients, as these are either at (1) the preclinical stage, or (2) the early developmental stage, or (3) the small-scale pilot trial stage regarding safety and initial efficacy. Finally, these innovative drugs have mainly targeted the virus, HBV. However, CHB is a pathological condition where the significant concerns are liver damage and complications such as LC and HCC. The studies on the mechanisms of these factors reveal that the host’s immune response in CHB patients plays cardinal roles underlying the genesis of viral persistence, liver damage, and HBV-related complications. Both innate immunity and adaptive immunity are induced improperly in the context of chronic HBV infection [17–22]. Furthermore, the impaired actions of different immune-regulatory cells such as myeloid-derived suppressor cells and antigen-presenting dendritic cells (DCs) have been detected in chronic HBV infection [23–27]. The aberrant interplay among HBV, immune regulators cells, and host immunity might be responsible for the impaired activation of cytotoxic T cells, T cell exhaustion, and reduced cytolytic activity of natural killer cells, allowing viral persistence and liver damage [28–30]. Immune therapy for CHB is an approach that is dedicated to alter the immune dysfunction leading to control of HBV replications, containment of liver damages and slowing the progression to LC and HCC. However, it is paramount interest to provide scientific logics to validate the rationale of immune therapy in CHB. The first, experiments in HBV transgenic mice (HBV TM) has shown that immune therapy by HBV-related antigens, HBV antigen-pulsed dendritic cells, and HBV DNA resulted in HBV DNA negativity, seroconversion of HBeAg, and production of anti-HBs [31–33]. The next, in vitro studies in CHB patients, it has been found that patients who control HBV DNA replication and contain liver damages for prolonged period harbor increased frequencies of HBV-antigen-specific immunocytes in the liver compared to those who cannot control HBV replication and liver damages [34]. Finally, in vivo studies in CHB patients with different immune therapies have documented that some of the immune therapies caused reduction of HBV replication and containment of liver damages at the end of treatment [35]. Now, the major challenges remain to ensure sustained effects of immune therapy in CHB patients.

The limitation of commercially available antiviral drugs and the mechanisms underlying the genesis of CHB opened a new and novel field of treatment and management of CHB patients by manipulating the host immune system, the immune therapy for CHB.

In this review, we will discuss different immune therapeutic approaches that have been undertaken during the last three decades to treat CHB patients. This will provide broad insights into the scopes and limitations of ongoing immune therapies for treating CHB patients. Ultimately, the present review will discuss optimizing proper evidence-based and effective immune therapeutic strategies for CHB, a delicate but achievable goal for treating CHB patients.

## Why commercially available antiviral drugs could not stand the test of time for treating CHB even though these drugs are endowed with potent antiviral potentialities?

As discussed before, as of today, mainly six antiviral drugs are recommended for treating CHB patients. However, satisfactory outcome could not be attained by treating CHB patients with these drugs, although these drugs have been used independently or in combination with other antiviral drugs. It is natural to ask why these drugs could not stand the test of time as potential therapeutic agents for managing CHB patients. To analyze these factors, we critically evaluated some fundamental aspects of kinetics of the development of antiviral drugs for CHB. None of these drugs were developed based on the life cycle of the HBV, the nature of pathogenesis of CHB, and the pathogenetic factors related to the progression of complications such as LC and HCC. They were primarily developed for other viral infections and later optimized for use in patients with CHB. For example, lamivudine was the first oral NUC approved for CHB patients in 1998. However, the drug had been approved for the human immune-deficiency virus (HIV) in 1996 [36]. HIV is a cytopathic virus, and the downregulation of replication of HIV represents a natural treatment modality. On the other hand, CHB is induced by a non-cytopathic virus, HBV. Moreover, unlike HIV, HBV infection leads to the production of cccDNA in the nucleus of the hepatocytes. In addition, chronic HBV infection is associated with the development of progressive hepatic fibrosis, which is not a common feature of the HIV infection. Thus, NUCs cannot be an ideal drug for CHB, an immune-mediated disease. Even then, some patients receiving NUCs exhibit improvement in the extent of liver damage and containment of progression to complications. These may be related to the restoration of immunity following the suppression of HBV by NUCs [37–39], but more studies are necessary to confirm this. Thus, the limitations of re-purposed antiviral drugs for the treatment of CHB are pretty evident, and the factors have been summarized in Table 1.

**Table 1:** Factors underlying the limitations of commercially available antiviral drugs for the treatment of CHB and in the context of achieving the target of “Elimination of Hepatitis by 2030”

Scientific limitations of IFN and NUCs	They are able to reduce or induce the negativity of replicating HBV DNA but unable to control cccDNA Repurposed drugs for CHB were not developed on the basis of the life cycle of HBV or mechanisms of pathogenesis of CHB Immune modulatory capacities of these drugs are insufficient and may not be purpose oriented They are unable to induce host immunity of proper quality and the intensity required for sustained control of HBV replication in CHB patients Their role in the containment of hepatic fibrosis and carcinogenesis is elusive
Practical limitation of NUCs	NUCs should be used for years or an infinite duration The usage of NUC is not patient friendly in developing countries, as lowering of HBV DNA caused by NUCs leads to the cessation of treatment in most resource-constrained countries. These patients ultimately develop rebound of HBV and severe liver damages Periodic assessment of HBV DNA, ALT, and liver fibrosis is difficult in many countries with a high number of CHB patients
“Elimination of hepatitis by 2030”	Most patients with CHB are living in developing countries Only 10% of CHB patients know their HBV infectivity in these countries Detecting the “Missing Million” is the target of HBV elimination by 2030 There is a need for the development of “Finite Therapy” to replace NUC-based therapeutic approaches for the attainment of “Elimination of Hepatitis by 2030” or even later in developing countries

## Concept and design of immune therapy as treatment modalities for CHB

The apparent limitations of antiviral drugs in CHB patients provide some essential tips regarding the designing of an effective treatment regimen for CHB. The ideal treatment strategy should have at least the following characteristic features.

First, immune therapeutic agents for CHB patients should be safe for human usage. Fundamentally, CHB is a pathological condition characterized by the inflammation of the liver (hepatitis), which is induced by aberrant host immunity against the virus. Thus, there always remains a possibility of exacerbation of hepatitis after the administration of immune therapeutic agents in CHB patients. Thus, an ideal immune therapeutic agent for CHB should control HBV replication in a non-cytotoxic manner and contain liver damage so that hepatic fibrosis may be arrested.

Next, innovative immune therapeutic agents should be designed based on scientific evidence. Preclinical studies should optimize the design of immune therapeutic agents. This is especially relevant because NUCs endowed with highly potent antiviral properties against HBV could not contain the extent of liver damage and the development of complications.

Finally, if possible, immune treatment regimen or any other innovative therapeutic should preferably be designed for offering a finite therapeutic option for CHB patients, as it is clear that “Elimination of Hepatitis by 2030” or even “Elimination of Hepatitis by 2050” cannot be materialized without treating the millions of CHB patients residing in developing and resource-constrained countries. The majority of CHB patients of these countries would not be able to follow long-term treatment approaches, such as treatment with NUCs.

## Immune therapeutic agents for the treatment of CHB patients

As shown in Table 2, several essential parameters must be fulfilled to develop an effective therapeutic regimen for CHB. However, the development process of immune therapy did not follow these suggestions. Due to this fundamental omission at the stage of initiation, there is no recommended immune therapeutic approach for CHB patients that received consensus from physicians and patients. It is still regarded as a developmental approach.

**Table 2:** Fundamental characteristics of evolving immune therapies against chronic hepatitis B

A. Features related to safety and pathogenesis potential of immune therapeutic agents	Safe for human usage Not induce liver damages Should not allow progressive hepatic fibrosis Should not be carcinogenic
B. Factors pertaining to scientific basis of development of immune therapies for chronic hepatitis B	Should be evidence-based Be endowed with antiviral and liver protecting capacities
C. Nature of immune therapies for chronic hepatitis B on the basis of duration of therapy	Should offer a finite or semi finite therapeutic option

**Fig. 1:**
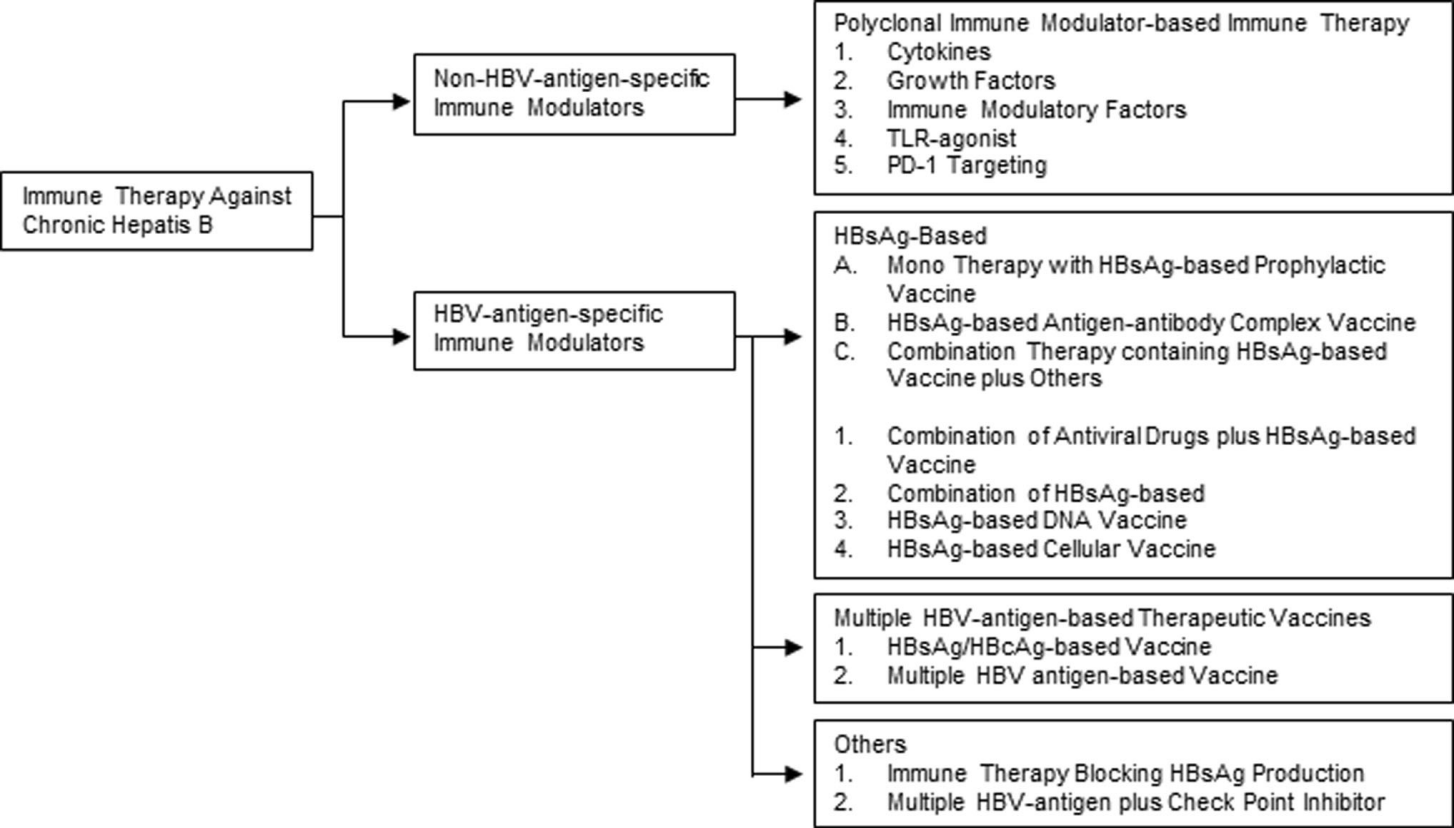
Immune therapeutics against chronic hepatitis B. Different immune therapeutic agents against chronic hepatitis B may be mainly divided into non-HBV-antigen-specific immune modulators and HBV-antigen-specific immune modulators. Different immune modulatory agents within these categories have been shown

Immune therapy for CHB patients was started in the mid-1990s. Investigators have used different immune modulators that can be divided into two main categories: (1) non-HBV-antigen-specific immune modulators and (2) HBV-antigen-specific immune modulators. As shown in Fig. 1, these two main groups of immune modulators may also be subdivided into various types on the basis of the nature of immune therapeutic modalities.

## HBV-antigen non-specific immune therapy using polyclonal immune modulators for the treatment of CHB

Several studies have revealed that HBV employs diverse, active strategies to evade innate immune responses, leading to specific types of immune suppression. The immune evasion and immune suppression by HBV are targeted via actions of different critical cells of innate immune systems, including dendritic cells, natural killer cells, T regulatory cells, and the interferon response signaling pathways [40–42]. Additionally, the production of cytokines is not properly induced by HBV. These improper inductions in innate immunity also result in impaired adaptive immunity in which the functions of almost all types of immunocytes are involved. Thus, the primary objective of immune therapy for CHB became the upregulation of host immunity in CHB patients by immune modulators with an assumption that this would reduce HBV replication and control HBV-induced liver damages.

To attain this goal, CHB patients were treated with polyclonal immune modulators since the early 1990s. Immune modulators such as interleukin (IL)-2, IL-12, granulocyte–macrophage colony-stimulating factor (GM-CSF), levamisole, thymus humoral factor-gamma 2, alpha-galactosylceramide, and thymosin alpha were administered to patients with CHB [43–51]. Use of these polyclonal immune modulators reduced HBV DNA and ALT in some patients, however, sustained effects of HBV replication or liver damagers have not been reported. Also, some of these agents were not safe in all CHB patients.

### Non-HBV-specific immune therapy using toll-like receptor (TLR) agonists against CHB

Thus, monotherapy of CHB patients with non-HBV-specific polyclonal immune modulators did not exhibit any promising outcome from 1993 to 2000. Human consumable cytokines and growth factors were mainly used during that period. With the advent of science, it has been shown that toll-like receptors (TLRs) represent the initial sensors of microbial infection, and stimulation of TLR by viruses may result in cellular and molecular events that induce various antiviral mediators [52, 53]. Thus, one of the notable modes of HBV suppression may be mediated and accelerated using TLR agonists. GS-9620 is a TLR-7 agonist, and CHB patients were treated with GS-9620 to assess if there is any therapeutic efficacy of GS-9620. Although the TLR-7 agonist showed potent efficacy in chimpanzees, therapeutic effects of this agent could not be documented in CHB patients [54, 55]. Another TLR-8 agonist is GS-9688. It showed immunogenicity in humans, but therapeutic efficacy on CHB patients is still inconclusive and should be shown by phase I/II or III clinical trials [56, 57].

### A bird’s eye view of safety efficacy of non-HBV-specific polyclonal immune modulators as therapeutic modalities for treating CHB patients

Since the early 1990s, patients with CHB have been treated with commercially available polyclonal immune modulators. The majority of these agents possessed the ability to upregulate various immune parameters. One of the major concerns about these agents' safety remains. Without a dose-rising study design, it appears to be safe in some trials. Different doses of these agents have not been used in the majority of cases. On the one hand, little information about safety concerns is available, and on the other hand, little information about efficacy with proper doses is available. The majority of the studies were pilot studies or phase I/II clinical trials. Even though some studies revealed initial encouraging findings, long-term follow-up data is not available. The natural question is why the follow-up study was not completed or long-term follow-up data were not published despite the encouraging initial results. There is a scarcity of data on phase III clinical trials with polyclonal immune modulators, making it difficult to compare them to other drugs used to treat CHB, such as IFN and NUCs. Finally, the mechanism of action of polyclonal immune modulators in CHB patients has not been studied. Also missing is information on the role of polyclonal immune modulators in the progression of chronic liver disease, as most studies with these modulators focused on HBV DNA. In addition, information from one point during therapy was usually provided. Because TLR agonist has now been used in phase-wise clinical trials [56, 57], there is reasonable hope for treating CHB patients as a combination therapy, study design should be optimized to show sustained effect of these agents.

## Immunotherapy targeting the HBV antigen-specific immune therapy for CHB patients

Patients with CHB have a weakened immune response to HBV, despite the fact that they are not immune-compromised in general. CHB patients who allow HBV replication and are unable to control liver damage have higher frequencies of non-antigen-specific immunocytes in the liver. These immunocytes may cause liver damage by inducing cytokines or growth factors, as well as polyclonal immunity. However, this type of immunity isn't enough to keep HBV replication under control indefinitely. CHB patients who control HBV replication and have no liver damage, on the other hand, have more HBV antigen-specific immunocytes [40, 41]. As a result, in the mid-1990s, a new field of immune therapeutic approaches emerged, focusing on the induction of adaptive or antigen-specific immunity in CHB patients using HBV-antigen-specific immune modulators.

HBV-antigen-specific immune therapy was initially started with a prophylactic HB vaccine containing HBsAg. The decision to begin HBsAg-based immune treatment in CHB patients was based on several factors. These are some of them:

1. Since the 1980s, an HBsAg-based prophylactic vaccine has been available all over the world. In healthy people, the safety and immunogenicity of prophylactic HBsAg-based vaccines have been optimized. As a result, there was no reason to be concerned about safety of HBsAg-based immune therapy in CHB patients.
2. Anti-HBs antibodies are not detected in almost all CHB patients' sera, though other HBV-related antibodies are. Anti-HBc is found in all chronic HBV-infected people, and anti-HBe is found in a significant number of CHB patients. Anti-HBs, on the other hand, have a significant impact on both healthy people and CHB patients. Anti-HBs antibodies present in vaccinated people provide lifelong protection against new HBV infection. Furthermore, developing anti-HBs in patients with chronic HBV infection is considered complete recovery from CHB status.
3. These ideas provided a moral and scientific foundation for using HBsAg-based immune therapy in CHB patients. Because HBsAg is a key component of the preventive vaccine, treating CHB patients with an HBsAg-based vaccine was referred to as "VACCINE THERAPY."

## HBsAg-based vaccines for HBV antigen-specific immune therapy in CHB patients

### Monotherapy with a CHB vaccine based on HBsAg

Pol et al. first reported HBsAg-based immunotherapy in CHB patients in 1994 [58], and various clinical trials with HBsAg-based vaccines in CHB patients followed. In CHB patients, HBsAg-based immune therapy was found to be safe. In addition, HBsAg vaccine therapy reduced HBV DNA, HBeAg negativity, and anti-HBe seroconversion in some CHB patients [59, 60]. However, the effects were not significant when compared to a control group of CHB patients, and the HBsAg-based vaccine did not provide long-term antiviral or liver protection in CHB patients. HBsAg-based vaccine therapy, like clinical trials with polyclonal immune modulators, was mostly pilot studies or small clinical trials. Furthermore, studies enrolling HBsAg-based vaccine therapy revealed significant heterogeneity in terms of HBsAg dose, treatment duration, and type of patients enrolled [58, 60]. Furthermore, because it was not included in the study protocols, the mechanism of action of HBsAg-based vaccine therapy in CHB patients has remained largely unknown. However, it became clear that HBsAg-based vaccine therapy as a mono-therapeutic agent for CHB patients would not stand the test of time.

### Antigen–antibody complex vaccine against CHB based on HBsAg

Immune therapies were planned to improve the therapeutic efficacy of HBsAg-based vaccines in CHB patients by changing the nature of the therapeutic vaccine, dose, or duration of treatment, and adopting significant changes in treatment design. In a pilot study and a well-planned phase IIb clinical trial, CHB patients were given a therapeutic vaccine that contained both HBsAg and hepatitis B immunoglobulin. The clinical outcome of the trials, however, was unsatisfactory [61–63].

### Combination therapy of antiviral drugs and an HBsAg-based vaccine is used

Initially, patients with high levels of HBV DNA in their sera were given the HBsAg-based vaccine. In addition, the majority of the patients in the early trials were HBeAg-positive. With the introduction of NUCs, it became possible to drastically reduce HBV DNA. In addition, the use of NUCs resulted in HBeAg negativity or anti-HBe seroconversion. In this scenario, it was assumed that an HBsAg-based therapeutic vaccine would be more effective in patients with CHB who had low HBV DNA levels, undetectable HBV DNA, were HBeAg-negative, or had anti-HBe antibodies. Based on these findings, a new field of immune therapy was established, in which HBsAg vaccine therapy was used in combination with antiviral drugs. The basic idea was that antiviral drugs would reduce HBV DNA and create an intrahepatic mucosal milieu that would allow immune therapy to work more effectively. Several studies of antiviral medications in combination with HBV antigen-based vaccines have been completed [64, 65]. Hoa et al. used a vaccine containing HBV Pre-S1, Pre-S2/S antigens and lamivudine in a randomized-controlled trial. Although the initial results of the lamivudine plus vaccine combination were promising, sustained effects could not be documented after 18 months when compared to lamivudine and vaccine recipients alone [66]. Even using an adjuvant in combination therapy with an antiviral drug and an HBsAg-based vaccine could not provide a long-term effect as a CHB treatment option. Even when used with antiviral drugs or in CHB patients with low HBV DNA, these trials show that the HBsAg-based vaccine is not committed to being a therapeutic agent of sufficient efficacy [67].

### DNA-based immune therapy for CHB that induces HBsAg

Different types of DNA vaccines are being tested in combination with antiviral drugs to treat CHB patients. Gordon et al. discovered that DNA vaccine had some beneficial effects in CHB patients who received NUCs [68]. The final analysis, however, revealed that DNA vaccination does not prevent relapse after NUCs are stopped. In a clinical trial, another candidate DNA vaccine, JNJ-64300535, was tested in combination with NUCs, but the DNA vaccine's efficacy was not proven [69].

### Immunotherapy for CHB patients using HBsAg-pulsed dendritic cells

Cell-based immune therapy for CHB patients has been developed using antigen-presenting dendritic cells (DC). To make HBsAg-pulsed DC, autologous DC was cultured with HBsAg. These were not only safe, but they also showed a reduction in HBV DNA and disease containment at the end of treatment. However, there are no follow-up data on the long-term efficacy and mechanism of action of cell-based vaccines [70–72].

## Epitope-based therapeutic vaccine therapy for the treatment of CHB patients

Due to the inherent limitations of the HBsAg-based vaccine, researchers turned their attention to immune therapy based on other HBV antigens, and epitope-based HBcAg was enrolled as a vaccine candidate. In CHB patients, a therapeutic vaccine containing an epitope HBV CD8 T-cell core epitope (Core18-27) with lipoprotein base was given. According to the findings, this vaccine was neither more immunogenic nor more effective in CHB patients [73].

## Multiple HBV-related antigens are used to treat CHB patients

Initially, vaccine therapy was primarily based on the use of HBsAg, as anti-HBs development was a treatment goal. However, inconclusive and frustrating experiences with HBsAg-based vaccine therapy, whether as individual therapy, or with NUCs, or with DNA vaccine, prompted the use of HBcAg as a component of HBV-antigen-based immune therapy. Simultaneously, data on the efficacy of HBcAg as a therapeutic option in preclinical trials has been accumulating. In addition, most studies on the pathogenesis of CHB in the 1990s focused primarily on immunological markers in the peripheral blood. In the late 1990s Maini et al. discovered that HBcAg-specific immunity in the liver may be a critical determinant of HBV replication control and liver damage management. In CHB patients, liver immunocytes were studied, and it was discovered that HBcAg-specific immunocytes were linked to HBV replication control and liver damage containment [40].

### Immunotherapy for CHB based on HBsAg/HBcAg

In Cuba, NASVAC, a therapeutic vaccine containing both HBsAg and HBcAg, was developed. This vaccine was able to activate both systemic and mucosal immunity due to its ability to be administered via parental and nasal routes. It was also discovered that nasal administration induced broad-based immunity in various mucosal compartments [74]. A phase I/II clinical trial of NASVAC was completed after the safety and immunogenicity studies of NASVAC in normal control subjects [75]. The results of phase I/II show that NASVAC has antiviral and liver-protective properties both at the end of treatment (EOT) and 48 weeks after EOT [76]. Following this, a phase III clinical trial in 160 CHB patients compared the safety and efficacy of NASVAC to pegylated interferon. NASVAC had a superior safety profile, significantly higher antiviral capacities, and dominant liver-protective capacity than patients receiving pegylated IFN at the end of treatment (EOT) and 24-weeks after EOT [77]. The NASVAC study group is one of the few that has published NASVAC patient follow-up data. A significant number of NASVAC recipients retained their antiviral and liver-protective status two and three years after EOT [78, 79]. Although NASVAC's initial trial did not examine its effect on quantitative HBsAg (qHBsAg) in CHB patients, NASVAC has recently demonstrated its effect on qHBsAg in an ongoing Japanese trial in which NASVAC has been administered to CHB patients along with NUCs [80].

### CHB vaccines based on multiple HB antigens

The biological product GS-4774 is derived from yeast. It expressed antigens for X, Core, Surface, and Polymerase. HBV X, S, and core antigens have been engineered into this. GS-4774 was tested in patients taking antiviral drugs who had low levels of HBV DNA in a phase II study. Although the immune therapeutic agent was well tolerated, there was no evidence of its therapeutic efficacy in CHB patients [81–83]. Although the study with GS-4774, which is made up of multiple HBV antigens, did not show a positive result in NUC-treated patients, it has yet to be evaluated in treatment-naive CHB patients. As a result, multiple HBV-related antigens appear to have the potential to be effective immune therapy for CHB, either alone or in combination with other agents.

## Checkpoint inhibitor-based immunotherapy

Immune checkpoints are meant to keep the immune system from becoming overworked, but they can also be linked to immune anomalies in some cases. Checkpoint proteins have been shown to be inhibitory in nature in CHB, and they may block immune induction or immunocyte activity. In HBeAg-negative CHB patients, a phase I pilot study compared anti-PD-1 (nivolumab) treatment with or without GS-4774 (therapeutic vaccine) treatment. Only a small percentage of patients had reduced HBsAg titers, with one of them having total and persistent HBsAg loss [84].

## Immunotherapy aimed at preventing the production of HBsAg

The induction and maintenance of HBsAg-specific immunity are hampered by the presence of large amounts of HBsAg as sub-viral particles. As a result, a therapeutic regimen for reducing HBsAg in the sera was developed. REP 2139, a nucleic acid polymer, is a potent blocker of the release of HBsAg from infective hepatocytes. In an open-label, randomized phase II study of HBeAg-negative CHB patients, the safety and efficacy of REP 2139 in combination with tenofovir and Peg-IFN were investigated. REP2139 was found to have some therapeutic efficacy, but more research is needed to determine the true implications of this agent as an innovative immune therapy [85–87].

## Possible mechanism of action of immune therapies against chronic hepatitis B

**Fig. 2:**
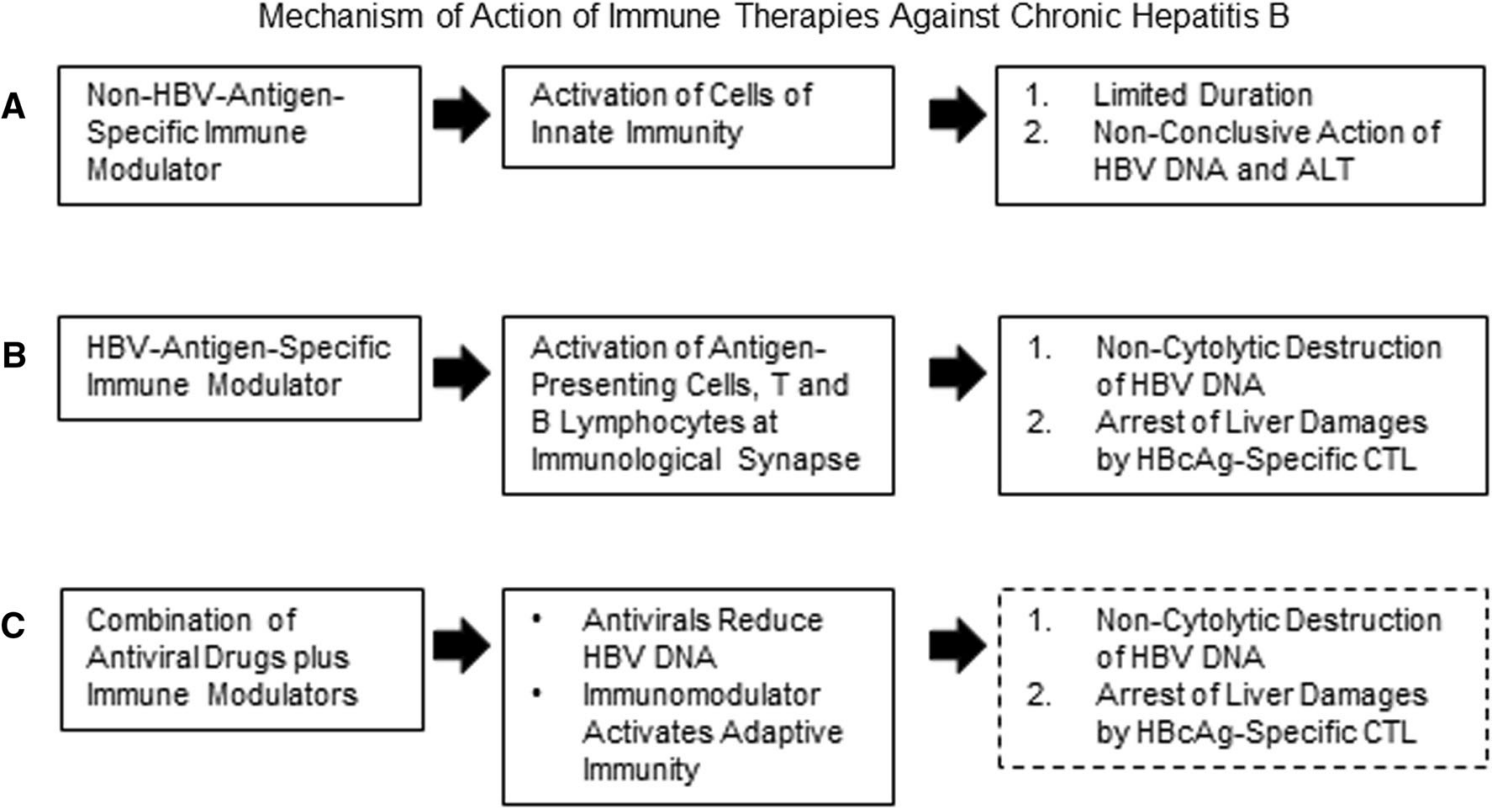
Mechanism of action of immune therapies against chronic hepatitis B. **A** Non-antigen-specific immune modulators usually activates cells of the innate immunity and these are of limited duration. Accordingly, independent usage of these agents is usually unable to contain HBV replication or control liver damage. **B** HBV-antigen-specific immune modulators activate antigen-presenting cells and induces antigen-specific immunocytes, these are usually long-lasting. These HBV-specific immunocytes are capable of non-cytolytic containment of HBV replication. If the HBV-antigen-specific immune modulators contain HBcAg, it is highly plausible that HBcAg-specific CTL would control liver damages, as shown in CHB patients [40]. **C** The cellular mechanisms underlying usage of antiviral drugs and immune modulators have not elucidated in CHB patients and this remains an area of investigation. It may be postulated that antiviral drug would reduce the levels of HBV DNA. The immune regulatory role of combination therapy would depend on the nature of the immune therapeutic agents

On the one hand, different patients with CHB are characterized by variable levels of HBV DNA and ALT in the sera with considerable heterogeneity regarding the progression of hepatic fibrosis in the liver. On the other hand, the immune therapeutic strategies are endowed with significant diversities regarding the nature of the immune therapeutic agent as well as the design of the trials. Accordingly, it is highly versatile to explain the mechanism of action of immune therapeutics in CHB patients. As shown in Fig. 2, non-antigen-specific immune modulators usually upregulate the activities of the cells of the innate immunity [40, 46]. However, these mediators either have insignificant effects on containment of HBV replication and liver damage, possible due to their inaction to induce HBV-specific immunity. On the other hand, if the levels of HBV-antigen-specific immunity are purpose oriented and of significant magnitudes, these are supposed to induce non-cytopathic destruction of HBV DNA [40]. This process may also favor containment of liver damages as HBcAg-specific immunity seems to be endowed with this property. Finally, in the context of combination of antiviral drugs and HBV-antigens-specific immunity therapy, antiviral drugs would reduce HBV replication and immune modulators may induce host immunity, however, the cellular and molecular events are yet to be checked in CHB patients more elaborately.

## Conclusion

During the last 3 decades, patients with CHB have been treated with a variety of immune therapeutic approaches. Immunotherapy against CHB appears to be a valid and innovative therapeutic concept, based on data from hundreds of pilot studies, clinical trials, and current scientific knowledge. The most significant contribution of various trials involving immune therapy for CHB is the generally excellent safety profiles of immune intervention in CHB patients. Immune therapeutic modalities, regardless of their nature (polyclonal immune modulators, HBV-antigen-based vaccines, combinations of antiviral drugs and vaccines, cell-based vaccines, or other types of immune therapeutic agents with TLR agonists or checkpoint inhibitors), were mostly safe for CHB patients with minor exceptions. The first and most important hurdle in the installation of immune therapy for CHB has been overcome. The next step is to develop immune therapeutic strategies based on the information gathered. Polyclonal immune modulators and only HBsAg-based vaccine therapy appear to have a short shelf life as new CHB therapeutics, though future studies with massive dose, duration, and study design changes may reveal their true potential. The real impact of immune checkpoint inhibitors, TLR agonists, and other newly developed polyclonal immune modulators as partners of immune therapeutics with other agents should be given special consideration. Multiple HBV-antigen-based immune therapeutics (HBsAg/HBcAg, all HBV antigens, epitope-based vaccine) may produce promising results when used alone or in combination with other drugs (antiviral drugs or other immune modulators). However, the order in which immune modulators and antivirals should be used (antiviral drugs concurrently or followed by immune therapeutic agents, or immune therapy followed by antiviral drugs) remains a significant scientific puzzle, which should be resolved through clinical trials. More clinical trials are needed to optimize the use of immune modulators while considering the characteristics of the patients (naive versus NUC-treated, doses of combined antigens, duration of therapy, adjuvant use, and nature of therapy) (independent or combination). To summarize, we now have some safe immune therapeutic options for CHB patients, and some of them have demonstrated significant antiviral and liver-protective properties. The development of an acceptable immune therapeutic option for CHB patients would be aided by the proper design of therapeutic intervention with these immune therapeutic modalities in different populations.
